# Re-examining hidden fitness: Female preferences for long-path songs in zebra finches

**DOI:** 10.1371/journal.pone.0343886

**Published:** 2026-03-05

**Authors:** Prateek K. Sahu, Moriah J. Deimeke, Alexandra D. Garcia, Katharine H. Stenstrom, Sarah M.L. Smeltz, Yu Wang, Julian Ratch, Christopher B. Sturdy

**Affiliations:** 1 Department of Psychology, University of Alberta, Edmonton, Canada; 2 Department of Computing Science, University of Alberta, Edmonton, Canada; 3 Neuroscience and Mental Health Institute, University of Alberta, Edmonton, Canada; Newcastle University, UNITED KINGDOM OF GREAT BRITAIN AND NORTHERN IRELAND

## Abstract

Female songbirds can evaluate male quality from secondary sexual characteristics such as songs. Studies have shown mixed findings on which acoustic features contribute to song attractiveness. In a 2024 study, Alam and colleagues introduced a holistic measure of song called “path length” and found that female zebra finches (*Taeniopygia guttata*) prefer long path length songs as compared to short path length songs. The original female-playback study was criticized for design limitations, notably its small sample size, and pseudoreplication. Here, we conducted a preregistered replication and extension study of the female preference for long vs. short path length songs. Our study will evaluate the robustness and generality of this preference.

## Introduction

Traits influencing mate choice are drivers of sexual selection. Much of the work in this field has focused on secondary sexual traits and the informational value and sensory modality of signals (such as visual or acoustic cues or multimodal cues) [1,2]. Many of these traits (such as plumage coloration, and elaborate song) can reflect male quality. The reliability of the signals depends on the cost associated with signals, modality, and contextual information [3–6]. Individual differences in sensory processing in females can also influence mate choice [7].

Birdsong has been widely studied as a model for understanding sexual selection [8]. Female songbirds can evaluate male songs to make an informed decision on mate choice [9], and their preferences for songs are often used as a proxy for mate choice [10–12]. Zebra finches (*Taeniopygia guttata*) sing a single song consisting of various syllables [13]. Song structure (such as duration, and repertoire size), and song performance (rate, and amplitude) can influence female mate preference [11]. Song complexity, which typically measures variability and stereotypy, has a weak relationship with mating success and depends on other biological and methodological factors [14]. Recent studies on zebra finches indicate low repeatability in female mate preferences, suggesting that these preferences are individual-specific [15,16]. One explanation can be the discrepancy between what information is conveyed by songs (such as individual identity or motor control) and how much is perceived by females in various contexts.

Rather than testing if isolated acoustic features of songs are attractive, one could examine if holistic measures of songs are attractive. Alam and colleagues [17] extracted path-length, a holistic measure of songs that can be an honest indicator of fitness in zebra finches. Though the study is not without criticism [18], holistic approaches remain promising [19]. The key question is not whether a single acoustic variable can predict preference, but how much of the variation in preference strength any given metric can explain. Here, we performed a robust replication and extension of the behavioural preference experiment based on the study by Alam and colleagues [17].

In 2024, Alam and colleagues [17] used over one million song syllables from 49 male zebra finches with the Deep Avian Network (DAN) pipeline for robust song analyses. They used the DAN pipeline to segment syllables automatically from the whole songs, and then measured acoustic features for each syllable along with the coefficient of spectrograms based on intensity of pixels. Uniform manifold approximation and projection (UMAP) was used for dimensional reduction from acoustic features to obtain two dimensions. With the UMAP algorithm, they obtained distinct clusters for similar-sounding syllables. Imitated (learned from tutor) and improvised (no tutor) song syllables showed clear visual segregation with stable cluster structures on the UMAP latent feature space. For each individual, Alam and colleagues calculated all possible minimum Euclidean distances among all the syllable clusters to obtain a holistic acoustic song measure called “path length”. The path length for each individual bird was stable across different UMAP embeddings, and the relative proportion of each bird’s contribution to the total path length was also consistent across different UMAP embeddings. Thus, UMAP embeddings and path length can provide a holistic representation of zebra finch songs. Distance measures can be used for song complexity indices based on spectrogram cross-correlation of song syllables [20]. Acoustic features like Wiener entropy and frequency modulation were strong predictors of pairwise syllable path length distances, and change in mean pitch of the syllable along with duration, peak frequency, goodness of pitch or amplitude were significant positive or negative predictors of the pairwise syllable path length distances. The differences in the path length in songs may be biologically relevant. Longer path length songs were more difficult to learn for the juvenile birds as measured through acoustic similarities. Generally, a short path length song means a song’s syllables are largely similar or similar syllables occur sequentially, whereas a long path length points to a wider diversity of syllable types [17]. Studies have shown that song measures (including repertoire size, consistency and variability of syllables, and song performance) influence female song preference [11]. Thus, the path length could capture some or all of these features, playing a crucial role in mate selection.

To understand the biological relevance of the path length in song syllables, Alam and colleagues performed a place preference experiment to test female song preferences. For stimuli, they obtained natural syllables from the syllable clusters and created three pairs of synthetic songs of long and short path length (total: six songs); each song consisted of five syllables separated by a 25ms gap of silence. Each pair of songs was contrasted in path length distance value; Pair 1: Moderate difference (~ 15), Pair 2: Largest difference (~ 30), Pair 3: Smallest difference (~ 6) (see Extended Data Fig. 7 from original article [17]; data points extracted with *WebPlotDigitizer*). The syllables that were used to create synthetic songs had similar acoustic features: entropy, frequency modulation, goodness of pitch, amplitude modulation, acoustic similarity, and duration (from visual inspection of box plots; see Extended Data Fig. 5e to 5j of original article [17]). 13 female zebra finches were tested in the place preference experiment; each bird received a pair of songs from the three pairs of synthetic songs (six birds: Pair 1, three birds: Pair 2, and four birds: Pair 3; see Source Data Fig. 3 of original article [17]). Alam and colleagues concluded that the female birds strongly preferred songs with longer path length to those with shorter path length.

Bulla and colleagues released a rebuttal preprint on the article by Alam and colleagues discussing several issues with the paper [18]. The issues include statistical artifacts in correlational analysis, reliability of the path-length metric, and limitations in experimental design. For the replication experiment, we focus on the female preference experiment. For the replication experiment, we will use Pair 2 (largest contrast) short vs. long path-length song as stimuli with a larger sample size in an operant (two-choice) preference experiment (See Table 1 for details). We re-analyzed the female place preference experimental data to help inform choices in translating the three-choice behavioural place preference test to a two-choice operant preference experiment. In the original study [17], Alam and colleagues used percentage time spent in the long path-length arm as a response variable to test if there was an effect of playback conditions (Pre vs. Trial vs. Post). Alam and colleagues did not account for the repeated measures error (ANOVA instead of using repeated measures ANOVA), so we re-analyzed the data with repeated measures ANOVA to obtain the effect sizes. We also ran repeated measures ANOVA with raw time spent on each arm (short, long, and home arm) as the response variable to estimate effect sizes in time spent on short vs. long path length song arms through playback conditions (raw time spent obtained from Extended Data Fig. 6 graph via *WebPlotDigitizer*). Generally, the effect sizes were moderate to large for most comparisons and a relatively small standard error (see Supplementary Materials S1 File for details). We used this information to make decisions on experimental design and analysis (see Supplementary Materials S1 File and Statistical analysis section).

**Table 1:** Summary of the original study, replication and extension study.

	Original Study	Replication & Extension
**Sample size**	13	22
**Preference test**	Place preference test with T maze	Operant preference test with IR perches
**Measurement**	Time spent	Perch triggers
**Stimuli**	3 pairs	1 pair from original study for replication & 20 new for extension
**Experimental design**	Each bird received one pair of stimuli out of three	Each bird received all stimuli in counter-balanced sessions
**Statistical analyses**	ANOVA	Bayesian hierarchical generalized linear mixed model

In addition to the replication, we will also perform an extension experiment with a larger stimulus set [18,21]: ten synthetic songs with short path lengths and ten with long path lengths. The raw syllables for these motifs will be extracted from a different archive of zebra finch recordings [22,23] rather than from the population sampled in the original study. Here, we plan to investigate whether path length as a structural property of song remains attractive. If song path length does play a role in mate preference, the extension experiment would provide a stronger, population-general test of this hypothesis. In sum, we will conduct two counter-balanced operant preference experiments: replication using the original authors’ stimuli and an extension using an independently constructed stimulus class.

## Methods

### Subjects

Twenty-two adult female zebra finches (*Taeniopygia guttata*) (at least 100 days old) will be used for both experiments. The birds were bred and raised at the University of Alberta, Edmonton, Canada, whose parent birds were supplied by a commercial breeder (Eastern Bird Supplies, Canada). Prior to the experiment, the birds were housed in an aviary (size: 172 cm × 166 cm × 74 cm) with many perches, *ad libitum* water, mixed seed (Hagen, Canada), and supplemented with greens (spinach, parsley) and vitamin water (Hagen, Canada) three times per week and spray millet (Hagen, Canada) once per week. The aviary housing room was kept on a 14:10 light:dark cycle with ~40% humidity and 24–25 °C temperature. The birds have previous experience with nest building material preference experiments but no experience with the current methodologies.

### Stimuli

We will use two sets of stimuli for the experiments: a replication set for the replication experiment and extension set for extension experiment.

Replication set: Two 5-syllable synthetic motifs with the largest path-length contrast (Pair 2) from the original study (Supplementary audio 3 & 4) [17] will be used for the replication experiment.

Extension set: We will construct 10 long and 10 short path-length 5-syllable synthetic motifs (20 total stimuli) following a similar procedure as described in [17]. Below we briefly describe the procedure.

### Syllable analysis for synthetic motifs

In the original study [17], Alam and colleagues used a Deep Avian Network (DAN) pipeline to automatically segment thousands of song syllables from whole songs with minimal manual annotations. Alam and colleagues validated this segmentation with a Siamese convolutional neural network (sCNN).

Briefly, a DAN pipeline works by detecting silences between the song syllables using amplitude thresholds and various filtering techniques to segment individual song syllables. For each segmented syllable, the pipeline extracts acoustic features including root mean square amplitude, entropy, pitch goodness, mean frequency, power spectrum density, and spectrogram pixel intensity. These features are then reduced to two dimensions using UMAP dimensionality reduction, creating embeddings that serve as training inputs for the convolutional neural network.

The sCNN uses the patterns from the acoustic features to classify the syllables and form clusters, which can be compared against manual segmentation for accuracy assessment. The original study showed that the DAN pipeline accurately segmented the syllables from the whole songs (see Extended Data Fig 1 from original study [17]). Together, the DAN-sCNN approach provides a robust and efficient method for segmenting hundreds of thousands of song syllables from large song datasets.

For our study, we will obtain ~160 songs produced by 6 male zebra finches (mean songs per individual bird: 20, range: 12–40) from a previously generated dataset from [22–24] to extract the song syllables. For our relatively small dataset, we will manually extract each syllable from songs using Praat software v 6.04.06 and save it as a*.wav* file. Every syllable file will be converted to a colour spectrogram image by a custom Python script derived from the DAN pipeline [17]. The script performs a Short-time Fourier Transform (STFT) on each syllable with consistent parameters (n_fft = 512, win_length = 256, hop_length = 8) and power spectra expressed in decibels. Each spectrogram will be plotted on a square canvas, stripped of axes, and with the same blue-white-red colormap that the original study used.

To obtain the feature matrix, each spectrogram image will be resized to 28 × 28 pixels, flattened, and scaled (0–1), producing a 2352 element vector (28 × 28 × 3). The feature matrix will be saved as a*.csv* file for downstream processing. Each feature matrix will be z-scored and projected into two dimensions with the UMAP algorithm (Uniform Manifold Approximation and Projection), which is typically used for dimension reduction and visualization of clusters [25]. The resulting coordinates (UMAP 1, UMAP 2) will be appended to the feature matrix. Visual inspection of the UMAP scatterplot can confirm whether syllables belong to the same syllable type and individuals form compact clusters, and syllables from different syllable types and individuals occupy distinct regions of the embedding space.

For each individual bird’s songs, we will calculate the geometric mean of the UMAP coordinates of all the syllables in a cluster. For example, for a bird with *k* distinct syllable clusters, there can be *k!* possible syllable orders. We will then compute the Euclidean distance between consecutive cluster centroids for each permutation and sum those distances to obtain a path length for every syllable ordering. There are criticisms of the reliability of UMAP as a clustering algorithm and its distance calculations among clusters [18,25]; therefore we will use both the HBDSCAN and *k-*Means clustering algorithm to validate our results. Additionally, we will use a variational autoencoder (VAE)-based method for clustering and path length calculations. VAE, an unsupervised neural network approach, outperforms traditional methods (with handpicked acoustic features) for quantifying variability and similarity of zebra finch tutor and pupil songs [26]. In the VAE method, spectrograms of song syllables will be used as input for clustering following the procedure by Goffinet and colleagues [26]. For consistency and robustness, we will compare the results across multiple distance metrics, i.e., Euclidian, cosine, Manhattan, and Chebyshev among syllable clusters. Ideally, all the methods should yield similar results; however, in case of large discrepancies, we will follow the methodological framework by Alam and colleagues.

To construct the playback stimuli, we will draw syllables from the whole syllable clusters where each synthetic motif consists of 5 syllables with a 25ms silence gap. The chosen syllables will have similar spectral and temporal acoustic features such as entropy, frequency modulation, acoustic similarity, goodness of pitch, amplitude modulation, and duration. A total of 20 stimuli, 10 long and 10 short path length synthetic motifs, will be created. If we are unable to create a sufficient number of new synthetic songs, we will use the syllables from the original study instead.

### Apparatus

An experimental cage of size: 36 × 25 × 40 cm (Rolf C. Hagen, Inc. Montreal, QC) will be placed on a table (height: ~ 90 cm) inside a sound attenuating chamber (117 × 120 × 200 cm; Industrial Acoustics Company, Bronx, NY). The cage will have two IR perches (IR Break Beam Sensors-3 mm LEDs, Adafruit Industries, USA) on either side and one regular perch in the middle of the cage. Two 8-ohm Dayton Audio PS95−8 3–1/2“ point source full-range speakers (frequency range: 110 Hz to 20 kHz, Dayton Audio, Springboro, USA) will be placed on either end of the experimental cage. The playbacks from the speakers and IR sensors will be controlled by a Raspberry Pi Zero 2 W (Raspberry Pi Foundation, UK) with HiFiBerry DAC+ Zero (Modul 9 GmbH, Switzerland) and a Cambridge A300 Integrated Amplifier (Cambridge Audio, UK).

### Experimental procedures

On the testing days, each bird will be transferred into the experimental cage inside the sound attenuating chamber and given at least 30 min for acclimation. Before the start of the playback session, baseline left-right preference will be determined where both of the IR perches will be active for 10 min without audio playback. If the bird triggered one perch more than the other (preference ratio ≥ 0.80), a second 10-min baseline will be run. Birds that still displayed the same bias after this second baseline will be retested on a later day and removed from the study if the bias persisted.

The playback session will last for 30 min for both replication and extension experiments. The playback duration is long enough to yield a minimum of ~100–120 responses (including interrupted trials) for most birds [27]. After this, during the subsequent post-playback, the IR perches will be active without audio playback for 10 min to measure if preference persisted. The 22 birds will be evenly split between two counter-balanced groups: one group (N = 11) will complete the replication experiment first, and the other group (N = 11) will complete the extension experiment. After a minimum period of seven days, every bird will be retested again with the alternate experiment. Across sessions, the order of the experiments, and the left/right assignment of the short- and long-path songs will be counter-balanced.

The audio playback system will be controlled by a Python script program that controls experimental parameters. During a trial session, when the bird sits on one of the IR perches for 2 sec, breaking the IR beam, the program will play one randomly selected audio stimulus. Each IR perch will be assigned to play a category of stimuli (e.g., perch 1: short path length songs; perch 2: long path length songs), which will be switched across the experiments to limit side bias. The inter-trial interval will be 5 sec. If a bird leaves the perch without listening to the full duration of the audio stimulus, the audio will keep playing to avoid popping and be counted as an interrupted trial. The program will log the time the trial was initiated, the number of audio playbacks on each perch, interrupted trials, and stimulus ID. The response data will be stored as a *CSV* file.

### Statistical analysis

Preference ratio will be calculated as long path-length song perch visits/ (long path-length song + short path-length song perch visits). The statistical tests will be performed in R v 4.4.1 [28]. Graphs will be plotted with base R, *ggplot2* package [29], and *bayesplot* package [30], and graphs and figures will be edited either in Inkscape v 1.4 or Adobe Illustrator v 28.7.1. Given the binary choice response data (short vs. long path length song preference), we will use a Bayesian hierarchical generalized linear mixed model (GLMM) using a binomial distribution with a logit link function to estimate the probability of song preference. The primary response variable is choice (coded 0 for short-path length song and 1 for long-path length song) with individual bird identity as a random effect to account for repeated measures within birds.

The base model structure will be:

choice ~ (1 | BirdID)

Additional models will incorporate fixed effects such as phase (baseline, playback, post-playback), experiment type (replication vs. extension), experimental session order (first vs. second session), and random effects such as time of the day, and specific stimulus ID in the extension experiment. Model selection will be guided by leave-one-out cross-validation (LOO) comparisons.

We followed the design analysis framework [31] to calculate Type S [sign] error (probability of an estimate being in the wrong direction) and Type M [magnitude] error (factor by which the magnitude of an effect might be overestimated). The original study [17] found that in the playback trial condition (out of 5 mins), females (N = 13) spent a 0.598 ± 0.23 proportion of time in the arm playing long path length songs, 0.25 ± 0.17 proportion of time in the short path length arm and 0.15 ± 0.13 proportion of time in the neutral home arm (Data manually extracted from the graph in Extended Data Fig. 6 using *WebPlotDigitizer*; see S1 Table). Considering two choices of long path length arm and short path length arm, the preference ratio would be 0.70 (0.598/ (0.598 + 0.25)). We examined power, Type S error, and Type M error for three hypothetical preference ratios for long path length songs: strong preference (pr = 0.70), moderate preference (pr = 0.65, and pr = 0.60), and weak preference (pr = 0.55).

We ran simulations of one of our planned hierarchical linear mixed models (22 birds × ≈120 responses, random intercepts and slopes) and obtained an expected maximum standard error SE ≈ 0.22 (min: 0.08, mean: 0.11) in 10000 scenarios. Using the *retrodesign* function [31], we obtained the power, Type S, and Type M error. For an expected strong to moderate preference (pr = 0.70 and pr = 0.65), our design achieved adequate power (> 0.8) with minimal Type S error (< 0.001) and minimal exaggeration Type M (<1.12), indicating reliable calculation of estimates. For hypothetical moderate to weak preference (pr = 0.60 and pr = 0.55), our design will not reliably detect an effect of the long path-length songs with minimal Type S error (< 0.01) but with exaggeration factor Type M (1.4 & 2.7) and low power (0.45 & 0.14) (See S3 Table and code). Overall, our study design can provide a reliable estimate of preference if we were to assume the original study’s claims.

### Ethics

All the procedures will be followed according to the Canadian Council on Animal Care (CCAC) Guidelines and Policies with approval from the Animal Care and Use Committee for Biosciences for the University of Alberta (AUP 1937), which is consistent with the Animal Care Committee Guidelines for the Use of Animals in Research. Throughout the experiment, the birds will have ad-libitum access to food and water. After testing, the birds will be returned to their aviary housing room.

## Results

Results will be completed upon completion of Stage 2 following data collection.

## Discussion

Discussion will be completed upon completion of Stage 2 following data collection.

## Supporting information

S1 File Re-analysis and results of original article by Alam et al. (2024). (DOCX)

S1 Table Shows raw time spent (sec) on each long (long path-length), short (short path- length), and home (neutral) arms during 300 sec pre-trial, trial and post-trial. (DOCX)

S2 Table Effect sizes for repeated Measures ANOVA for time spent on each arm during Pre-trial, Trial, and post-trial. (DOCX)

S3 Table Type M and Type S error and power for different hypothetical preference ratio and corresponding long-odds in logistic models. (DOCX)
