# Supplementary material for: Re-examining hidden fitness: Female preferences for long-path songs in zebra finches
Source: PLoS One. 2026 Mar 5;21(3):e0343886. doi: 10.1371/journal.pone.0343886 (PMC12962475; doi:10.1371/journal.pone.0343886)
Supplement: S1 File — (DOCX) [file pone.0343886.s001.docx]

**Supplementary**

**Re-analysis of original article results**

We re-analyzed the data from the female preference experiment of the original article. The purpose of these analyses was to obtain effect sizes and standard errors of the original experiment to make informed decisions for our experimental design. The statistical analyses were performed in JASP v 0.19.3 [1]. We reported relevant effect sizes and standard error below, see the end of the Supplementary for detailed ANOVA tables.

**S1 Table. Effect sizes and standard error for repeated measures ANOVA for percentage of time spent on long path length song arm**. Data (Source Data Fig. 3) from original study re-analyzed.

| Comparisons | Effect size | Standard Error |
| --- | --- | --- |
| Group (Pre-Trial-Post) | η² = 0.53 | range (6.19, 7.64) |
| Trial - Pre | d = 1.26 | 3.62 |
| Trial - Post | d = 0.82 | 6.56 |

**
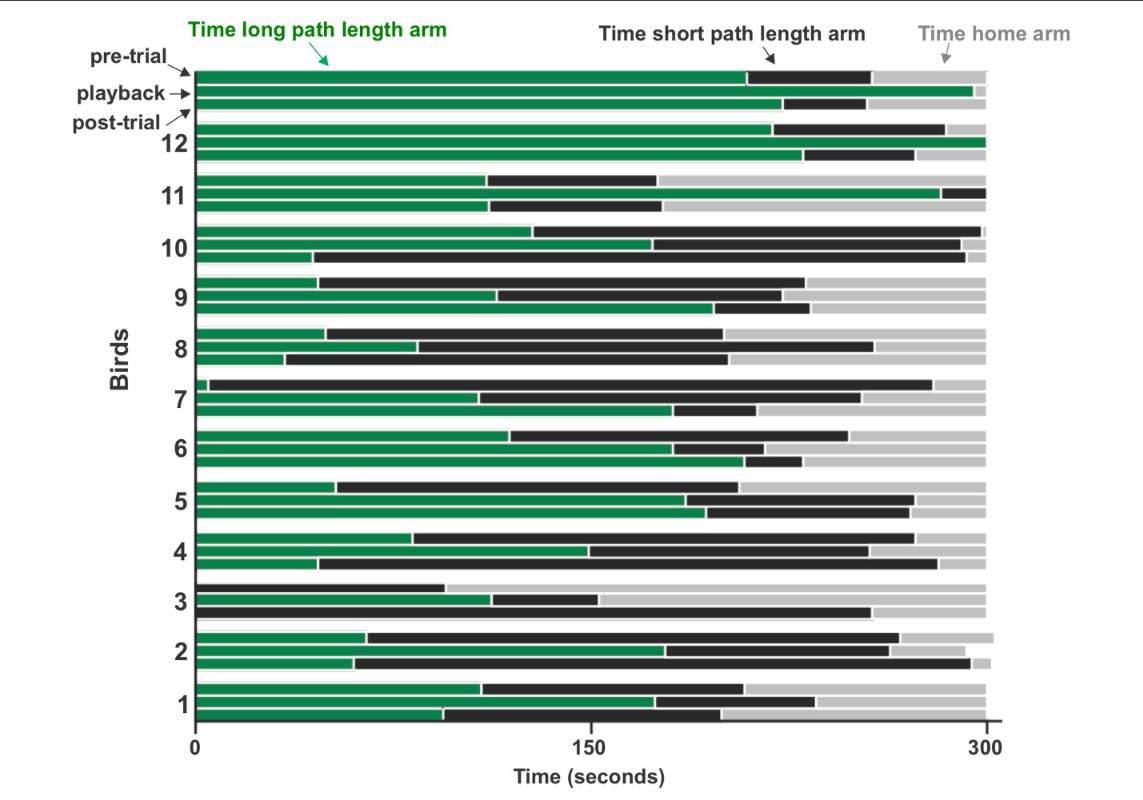
S1 Fig. Extended Data Fig. 6 from original article.** The time spent (sec) on each arm by each female zebra finch was extracted using *WebPlotDigitizer* (<https://automeris.io/>). See S2 Table for the extracted values of the graph.

**S2 Table. Shows raw time spent (sec) on each long (long path-length), short (short path-length), and home (neutral) arms during 300 sec pre-trial, trial and post-trial**. Also shows mean and standard deviation (SD) for proportion of time spent on each arm.

| **Bar # (ID Bird)** | **Pre** | | | **Trial** | | | **Post** | | |
| --- | --- | --- | --- | --- | --- | --- | --- | --- | --- |
|  | **Long** | **Short** | **Home** | **Long** | **Short** | **Home** | **Long** | **Short** | **Home** |
| Bar1 | 108.27 | 99.36 | 92.37 | 174.05 | 61.19 | 64.76 | 93.34 | 105.21 | 101.45 |
| Bar2 | 64.96 | 201.4 | 33.64 | 178.55 | 84.59 | 36.86 | 59.71 | 233.9 | 6.39 |
| Bar3 | 0 | 94.11 | 205.89 | 111.76 | 41.31 | 146.93 | 0 | 255.46 | 44.54 |
| Bar4 | 81.99 | 189.52 | 28.49 | 148.79 | 106.16 | 45.05 | 46.25 | 234.95 | 18.8 |
| Bar5 | 53.19 | 152.02 | 94.79 | 185.12 | 87.49 | 27.39 | 193.2 | 77.4 | 29.4 |
| Bar6 | 118.78 | 128.21 | 53.01 | 180.62 | 35.16 | 84.22 | 207.71 | 21.72 | 70.57 |
| Bar7 | 4.63 | 274.45 | 20.92 | 107.27 | 144.61 | 48.12 | 180.59 | 31.28 | 88.13 |
| Bar8 | 49.19 | 150.57 | 100.24 | 84.43 | 172.82 | 42.75 | 33.85 | 168.03 | 98.12 |
| Bar9 | 46.25 | 184.39 | 69.36 | 113.84 | 108.09 | 78.07 | 195.94 | 36.52 | 67.54 |
| Bar10 | 127.4 | 170.45 | 2.15 | 173.7 | 116.06 | 10.24 | 44.36 | 247.43 | 8.21 |
| Bar11 | 110.16 | 63.87 | 125.97 | 282.7 | 17.3 | 0 | 111 | 65.75 | 123.25 |
| Bar12 | 218.43 | 65.79 | 15.78 | 300 | 0 | 0 | 230.2 | 42.22 | 27.58 |
| Bar13 | 208.76 | 47.31 | 43.93 | 294.46 | 0 | 5.54 | 222.21 | 32.04 | 45.75 |
| Mean (SD) | 0.30 (0.21) | 0.46 (0.2) | 0.22 (0.19) | **0.59 (0.23)** | **0.24 (0.17)** | **0.15 (0.13)** | 0.41 (0.26) | 0.39 (0.30) | 0.18 (0.12) |

**S3 Table. Effect sizes for repeated Measures ANOVA for time spent on each arm during Pre-trial, Trial, and post-trial**. See the end of the Supplementary for detailed ANOVA table.

| Comparisons | Effect size | Standard Error | Standard Error (proportion scale) |
| --- | --- | --- | --- |
| Group (Pre-Trial-Post) | η² = 0.17 | range (10.6, 26.1) | range (0.02, 0.06) |
| Long arm-Pre vs short arm-Pre | d = -0.73 | 33.39 | 0.08 |
| Long arm-Trial vs short arm-Trial | d = 1.58 | 33.62 | 0.08 |
| Long arm-Post vs short arm-Post | d = 0.07 | 48 | 0.12 |

**Design analysis for experimental design**

We followed Gelman and Carlin’s framework [2] to make informed choices for sample size and interpretation of results which are influenced by expected true effect size and standard error. We considered the effect sizes from the original study and other conservative effect sizes with lower preference ratios for long path length songs. Standard errors are difficult to estimate prior to the experiment. We rescaled the standard errors from the original study to a proportion scale with the formula SE_new_​= (SE_old_ √(N_new_​/N_old_​))/300 (S3 Table). ​​But for our experiment, the standard error can be vastly different due to different experimental design and research equipment. We also simulated (10000 runs) possible standard errors for our experiment based on true population preference ratio of 0.6 (corresponding log-odds of 0.4) and ~ 120 trial response per individual bird. We calculated Type M and Type S errors based on the maximum standard error obtained from simulation to be more conservative (S4 Table). Keeping Type S error aside (as we predict a directional preference for long path-length songs), Type M errors suggest that with preference ratio of 0.70, the estimate would be 1% larger than true value and preference ratio lower than 0.65, the estimate would be 12% or larger than the true value. Note that any change in standard error can vastly alter these error calculations. For example, with sample size N=16, the expected maximum standard error from simulations was 0.24. Thus, making Type M error 1.02 for preference ratio 0.70 (power = 0.95) and 1.17 for preference ratio 0.65 (power = 0.73).

**S4 Table. Type M and Type S error and power for different hypothetical preference ratio and corresponding long-odds in logistic models.**

| Preference ratio | beta | Standard error | Power | Type S | Type M |
| --- | --- | --- | --- | --- | --- |
| 0.55 | 0.2006707 | 0.22 | 0.1493985 | 1.364637e-02 | 2.722482 |
| 0.60 | 0.4054651 | 0.22 | 0.4535250 | 1.576118e-04 | 1.466771 |
| 0.65 | 0.6190392 | 0.22 | 0.8034070 | 1.125215e-06 | 1.124541 |
| 0.70 | 0.8712224 | 0.22 | 0.9772573 | 1.646508e-09 | 1.013753 |

**Repeated measures ANOVA with percentage of time spent on long path length song arm (Re-analysis of original article results)**

| *Within Subjects Effects* | | | | | | | | | | | | | | | |
| --- | --- | --- | --- | --- | --- | --- | --- | --- | --- | --- | --- | --- | --- | --- | --- |
| Cases | | Sphericity Correction | | Sum of Squares | | df | | Mean Square | | F | | p | | η² | |
| RM Factor 1 |  | None |  | 6426.353 | ᵃ | 2.000 | ᵃ | 3213.177 | ᵃ | 13.586 | ᵃ | < .001 | ᵃ | 0.531 |  |
|  |  | Greenhouse-Geisser |  | 6426.353 |  | 1.397 |  | 4599.153 |  | 13.586 |  | < .001 |  | 0.531 |  |
| Residuals |  | None |  | 5676.338 |  | 24.000 |  | 236.514 |  |  |  |  |  |  |  |
|  |  | Greenhouse-Geisser |  | 5676.338 |  | 16.767 |  | 338.532 |  |  |  |  |  |  |  |
|  | | | | | | | | | | | | | | | |
| Note.  Type III Sum of Squares | | | | | | | | | | | | | | | |
| ᵃ Mauchly's test of sphericity indicates that the assumption of sphericity is violated (p < .05). | | | | | | | | | | | | | | | |

| *Between Subjects Effects* | | | | | | | | | | | |
| --- | --- | --- | --- | --- | --- | --- | --- | --- | --- | --- | --- |
| Cases | | Sum of Squares | | df | | Mean Square | | F | | p | |
| Residuals |  | 15873.687 |  | 12 |  | 1322.807 |  |  |  |  |  |
|  | | | | | | | | | | | |
| Note.  Type III Sum of Squares | | | | | | | | | | | |

| *Descriptives* | | | | | | | | | | | |
| --- | --- | --- | --- | --- | --- | --- | --- | --- | --- | --- | --- |
| RM Factor 1 | | N | | Mean | | SD | | SE | | Coefficient of variation | |
| Pre |  | 13 |  | 30.745 |  | 22.345 |  | 6.197 |  | 0.727 |  |
| Trial |  | 13 |  | 61.718 |  | 23.169 |  | 6.426 |  | 0.375 |  |
| Post |  | 13 |  | 41.537 |  | 27.563 |  | 7.645 |  | 0.664 |  |
|  | | | | | | | | | | | |

| *Post-hoc tests*  *Post Hoc Comparisons - RM Factor 1* | | | | | | | | | | | | | | | | | | | | | | | |
| --- | --- | --- | --- | --- | --- | --- | --- | --- | --- | --- | --- | --- | --- | --- | --- | --- | --- | --- | --- | --- | --- | --- | --- |
|  | | | | | | 95% CI for Mean Difference | | | |  | | | | | | | | 95% CI for Cohen's d | | | |  | |
|  | |  | | Mean Difference | | Lower | | Upper | | SE | | df | | t | | Cohen's d | | Lower | | Upper | | p_tukey_ | |
| Pre |  | Trial |  | -30.972 |  | -41.057 |  | -20.888 |  | 3.628 |  | 12 |  | -8.537 |  | -1.266 |  | -2.094 |  | -0.438 |  | . |  |
|  |  | Post |  | -10.792 |  | -31.004 |  | 9.421 |  | 7.272 |  | 12 |  | -1.484 |  | -0.441 |  | -1.304 |  | 0.422 |  | . |  |
| Trial |  | Post |  | 20.181 |  | 1.930 |  | 38.431 |  | 6.566 |  | 12 |  | 3.073 |  | 0.825 |  | -0.056 |  | 1.705 |  | . |  |
|  | | | | | | | | | | | | | | | | | | | | | | | |
| Note.  P-value and confidence intervals adjusted for comparing a family of 3 estimates (confidence intervals corrected using the bonferroni method). | | | | | | | | | | | | | | | | | | | | | | | |
| Note.  Tukey corrected p-values are not appropriate for repeated measures post-hoc tests (Maxwell, 1980; Field, 2012). | | | | | | | | | | | | | | | | | | | | | | | |

**Repeated measures ANOVA with raw time spent on each arm (New analysis of original article results)**

| *Within Subjects Effects* | | | | | | | | | | | | | | | |
| --- | --- | --- | --- | --- | --- | --- | --- | --- | --- | --- | --- | --- | --- | --- | --- |
| Cases | | Sphericity Correction | | Sum of Squares | | df | | Mean Square | | F | | p | | η² | |
| group |  | Greenhouse-Geisser |  | 118498.661 |  | 1.492 |  | 79415.426 |  | 4.764 |  | 0.030 |  | 0.177 |  |
| Residuals |  | Greenhouse-Geisser |  | 298486.836 |  | 17.906 |  | 16669.991 |  |  |  |  |  |  |  |
| group ✻ testing |  | Greenhouse-Geisser |  | 83526.610 |  | 1.877 |  | 44507.162 |  | 5.891 |  | 0.010 |  | 0.125 |  |
| Residuals |  | Greenhouse-Geisser |  | 170143.122 |  | 22.520 |  | 7555.065 |  |  |  |  |  |  |  |
|  | | | | | | | | | | | | | | | |
| Note.  Sphericity corrections not available for factors with 2 levels. | | | | | | | | | | | | | | | |
| Note.  Type III Sum of Squares | | | | | | | | | | | | | | | |
| ᵃ Mauchly's test of sphericity indicates that the assumption of sphericity is violated (p < .05). | | | | | | | | | | | | | | | |

| *Between Subjects Effects* | | | | | | | | | | | |
| --- | --- | --- | --- | --- | --- | --- | --- | --- | --- | --- | --- |
| Cases | | Sum of Squares | | df | | Mean Square | | F | | p | |
| Residuals |  | 1.026×10^-5^ |  | 12 |  | 8.547×10^-7^ |  |  |  |  |  |
|  | | | | | | | | | | | |
| Note.  Type III Sum of Squares | | | | | | | | | | | |

| *Descriptives* | | | | | | | | | | | | | |
| --- | --- | --- | --- | --- | --- | --- | --- | --- | --- | --- | --- | --- | --- |
| group | | testing | | N | | Mean | | SD | | SE | | Coefficient of variation | |
| long |  | pre |  | 13 |  | 91.693 |  | 67.418 |  | 18.698 |  | 0.735 |  |
|  |  | play |  | 13 |  | 179.638 |  | 72.216 |  | 20.029 |  | 0.402 |  |
|  |  | post |  | 13 |  | 124.489 |  | 82.778 |  | 22.959 |  | 0.665 |  |
| short |  | pre |  | 13 |  | 140.112 |  | 65.348 |  | 18.124 |  | 0.466 |  |
|  |  | play |  | 13 |  | 74.983 |  | 54.701 |  | 15.171 |  | 0.730 |  |
|  |  | post |  | 13 |  | 119.377 |  | 94.137 |  | 26.109 |  | 0.789 |  |
| home |  | pre |  | 13 |  | 68.195 |  | 55.940 |  | 15.515 |  | 0.820 |  |
|  |  | play |  | 13 |  | 45.379 |  | 41.449 |  | 11.496 |  | 0.913 |  |
|  |  | post |  | 13 |  | 56.133 |  | 38.319 |  | 10.628 |  | 0.683 |  |
|  | | | | | | | | | | | | | |

| *Test of Sphericity* | | | | | | | | | | | | | | | |
| --- | --- | --- | --- | --- | --- | --- | --- | --- | --- | --- | --- | --- | --- | --- | --- |
|  | | Mauchly's W | | Approx. Χ² | | df | | p-value | | Greenhouse-Geisser ε | | Huynh-Feldt ε | | Lower Bound ε | |
| group |  | 0.660 |  | 4.577 |  | 2 |  | 0.101 |  | 0.746 |  | 0.828 |  | 0.500 |  |
| group ✻ testing |  | 0.134 |  | 20.901 |  | 9 |  | 0.014 |  | 0.469 |  | 0.553 |  | 0.250 |  |
|  | | | | | | | | | | | | | | | |

| *Post Hoc tests*  *Post Hoc Comparisons - group* | | | | | | | | | | | | | | | |
| --- | --- | --- | --- | --- | --- | --- | --- | --- | --- | --- | --- | --- | --- | --- | --- |
|  | |  | | Mean Difference | | SE | | df | | t | | Cohen's d | | p_holm_ | |
| long |  | short |  | 20.449 |  | 31.470 |  | 12 |  | 0.650 |  | 0.310 |  | 0.528 |  |
|  |  | home |  | 75.371 |  | 23.758 |  | 12 |  | 3.172 |  | 1.144 |  | 0.024 |  |
| short |  | home |  | 54.921 |  | 18.935 |  | 12 |  | 2.900 |  | 0.834 |  | 0.027 |  |
|  | | | | | | | | | | | | | | | |
| Note.  P-value adjusted for comparing a family of 3 estimates. | | | | | | | | | | | | | | | |
| Note.  Results are averaged over the levels of: testing   \| *Post Hoc Comparisons - testing* \| \| \| \| \| \| \| \| \| \| \| \| \| \| \| \| \| --- \| --- \| --- \| --- \| --- \| --- \| --- \| --- \| --- \| --- \| --- \| --- \| --- \| --- \| --- \| --- \| \|  \| \|  \| \| Mean Difference \| \| SE \| \| df \| \| t \| \| Cohen's d \| \| p_holm_ \| \| \| pre \|  \| play \|  \| -2.132×10^-14^ \|  \| NaN \|  \| 12 \|  \| NaN \|  \| -1.332×10^-15^ \|  \| NaN \|  \| \|  \|  \| post \|  \| 2.564×10^-4^ \|  \| 2.564×10^-4^ \|  \| 12 \|  \| 1.000 \|  \| 3.893×10^-6^ \|  \| 0.674 \|  \| \| play \|  \| post \|  \| 2.564×10^-4^ \|  \| 2.564×10^-4^ \|  \| 12 \|  \| 1.000 \|  \| 3.893×10^-6^ \|  \| 0.674 \|  \| \|  \| \| \| \| \| \| \| \| \| \| \| \| \| \| \| \| \| Note.  P-value adjusted for comparing a family of 2 estimates. \| \| \| \| \| \| \| \| \| \| \| \| \| \| \| \| \| Note.  Results are averaged over the levels of: group \| \| \| \| \| \| \| \| \| \| \| \| \| \| \| \| | | | | | | | | | | | | | | | |

| *Post Hoc Comparisons - group ✻ testing* | | | | | | | | | | | | | | | |
| --- | --- | --- | --- | --- | --- | --- | --- | --- | --- | --- | --- | --- | --- | --- | --- |
|  | |  | | Mean Difference | | SE | | df | | t | | Cohen's d | | p_holm_ | |
| long, pre |  | short, pre |  | -48.418 |  | 33.399 |  | 12 |  | -1.450 |  | -0.735 |  | 1.000 |  |
|  |  | home, pre |  | 23.498 |  | 29.193 |  | 12 |  | 0.805 |  | 0.357 |  | 1.000 |  |
|  |  | long, play |  | -87.945 |  | 10.477 |  | 12 |  | -8.394 |  | -1.335 |  | < .001 |  |
|  |  | short, play |  | 16.710 |  | 30.820 |  | 12 |  | 0.542 |  | 0.254 |  | 1.000 |  |
|  |  | home, play |  | 46.314 |  | 27.436 |  | 12 |  | 1.688 |  | 0.703 |  | 1.000 |  |
|  |  | long, post |  | -32.796 |  | 22.054 |  | 12 |  | -1.487 |  | -0.498 |  | 1.000 |  |
|  |  | short, post |  | -27.684 |  | 36.763 |  | 12 |  | -0.753 |  | -0.420 |  | 1.000 |  |
|  |  | home, post |  | 35.560 |  | 23.080 |  | 12 |  | 1.541 |  | 0.540 |  | 1.000 |  |
| short, pre |  | home, pre |  | 71.916 |  | 28.085 |  | 12 |  | 2.561 |  | 1.092 |  | 0.624 |  |
|  |  | long, play |  | -39.526 |  | 35.150 |  | 12 |  | -1.125 |  | -0.600 |  | 1.000 |  |
|  |  | short, play |  | 65.128 |  | 10.557 |  | 12 |  | 6.169 |  | 0.989 |  | 0.002 |  |
|  |  | home, play |  | 94.732 |  | 20.049 |  | 12 |  | 4.725 |  | 1.438 |  | 0.016 |  |
|  |  | long, post |  | 15.622 |  | 31.281 |  | 12 |  | 0.499 |  | 0.237 |  | 1.000 |  |
|  |  | short, post |  | 20.735 |  | 28.704 |  | 12 |  | 0.722 |  | 0.315 |  | 1.000 |  |
|  |  | home, post |  | 83.978 |  | 22.467 |  | 12 |  | 3.738 |  | 1.275 |  | 0.085 |  |
| home, pre |  | long, play |  | -111.442 |  | 27.876 |  | 12 |  | -3.998 |  | -1.692 |  | 0.055 |  |
|  |  | short, play |  | -6.788 |  | 23.410 |  | 12 |  | -0.290 |  | -0.103 |  | 1.000 |  |
|  |  | home, play |  | 22.816 |  | 12.672 |  | 12 |  | 1.801 |  | 0.346 |  | 1.000 |  |
|  |  | long, post |  | -56.294 |  | 32.175 |  | 12 |  | -1.750 |  | -0.855 |  | 1.000 |  |
|  |  | short, post |  | -51.182 |  | 28.078 |  | 12 |  | -1.823 |  | -0.777 |  | 1.000 |  |
|  |  | home, post |  | 12.062 |  | 14.840 |  | 12 |  | 0.813 |  | 0.183 |  | 1.000 |  |
| long, play |  | short, play |  | 104.655 |  | 33.623 |  | 12 |  | 3.113 |  | 1.589 |  | 0.260 |  |
|  |  | home, play |  | 134.258 |  | 28.922 |  | 12 |  | 4.642 |  | 2.038 |  | 0.018 |  |
|  |  | long, post |  | 55.148 |  | 22.055 |  | 12 |  | 2.501 |  | 0.837 |  | 0.669 |  |
|  |  | short, post |  | 60.261 |  | 38.571 |  | 12 |  | 1.562 |  | 0.915 |  | 1.000 |  |
|  |  | home, post |  | 123.505 |  | 23.475 |  | 12 |  | 5.261 |  | 1.875 |  | 0.007 |  |
| short, play |  | home, play |  | 29.604 |  | 17.986 |  | 12 |  | 1.646 |  | 0.449 |  | 1.000 |  |
|  |  | long, post |  | -49.506 |  | 32.091 |  | 12 |  | -1.543 |  | -0.752 |  | 1.000 |  |
|  |  | short, post |  | -44.394 |  | 25.538 |  | 12 |  | -1.738 |  | -0.674 |  | 1.000 |  |
|  |  | home, post |  | 18.850 |  | 18.172 |  | 12 |  | 1.037 |  | 0.286 |  | 1.000 |  |
| home, play |  | long, post |  | -79.110 |  | 28.785 |  | 12 |  | -2.748 |  | -1.201 |  | 0.459 |  |
|  |  | short, post |  | -73.998 |  | 25.857 |  | 12 |  | -2.862 |  | -1.123 |  | 0.401 |  |
|  |  | home, post |  | -10.754 |  | 14.868 |  | 12 |  | -0.723 |  | -0.163 |  | 1.000 |  |
| long, post |  | short, post |  | 5.112 |  | 48.006 |  | 12 |  | 0.106 |  | 0.078 |  | 1.000 |  |
|  |  | home, post |  | 68.356 |  | 24.463 |  | 12 |  | 2.794 |  | 1.038 |  | 0.438 |  |
| short, post |  | home, post |  | 63.244 |  | 32.590 |  | 12 |  | 1.941 |  | 0.960 |  | 1.000 |  |
|  | | | | | | | | | | | | | | | |
| Note.  P-value adjusted for comparing a family of 36 estimates. | | | | | | | | | | | | | | | |

**R Code**

**1. Estimate standard error for our study design**

library(data.table)

library(lme4)

set.seed(754) # Load packages and set seed for reproducibility

## -----------------------------------------------------------------

# define study constants

n_birds <- 22 # number of birds

trials <- 120 # average trials per bird

beta <- 0.405 # true population log-odds (≈ 60 % pref)

sd_int <- 0.6 # SD of random intercepts

sd_slope <- 0.4 # SD of random slopes; both are conservative

## -----------------------------------------------------------------

## The below code simulates the standard error of the estimated slope with one of the generalized linear mixed model

n_rep <- 10000

SEs <- replicate(n_rep, {

DT <- data.table::CJ(bird = factor(1:22), trial = 1:120)

int_b <- rnorm(22, 0, 0.6)

slp_b <- rnorm(22, 0, 0.4)

DT[, long := rbinom(.N, 1, .5)]

DT[, eta := int_b[as.integer(bird)] +

(0.405 + slp_b[as.integer(bird)]) * long]

DT[, choice := rbinom(.N, 1, plogis(eta))]

fit <- lme4::glmer(choice ~ long + (1+long|bird),

DT, binomial, nAGQ = 0,

control = lme4::glmerControl(optimizer="bobyqa"))

sqrt(vcov(fit)[2,2])

})

summary(SEs)

**2. Estimate Type M and Type S error**

library(retrodesign)

library(data.table)

set.seed(754) # Load packages and set seed for reproducibility

p_vec <- c(0.55, 0.60, 0.65, 0.705) #potential p values

beta <- log(p_vec / (1 - p_vec)) # beta for logistic regression

se_beta <-0.22 # one of the standard error value

## Calculate power and error rate for each effect size

res_dt <- data.table(preference = p_vec, beta, se = se_beta)

res_dt[, c("power", "type_s", "type_m") :=

{gc <- retrodesign(beta, se);

list(gc$power, gc$type_s, gc$type_m)},

by = 1:nrow(res_dt)]

print(res_dt)

**References**

1. JASP Team. JASP (version 0.19.3) [computer software] [Internet]. 2025. Available from: https://jasp-stats.org/

2. Gelman A, Carlin J. Beyond Power Calculations: Assessing Type S (Sign) and Type M (Magnitude) Errors. Perspect Psychol Sci. 2014 Nov;9(6):641–51.
