# Supplementary material for: Re-examining hidden fitness: Female preferences for long-path songs in zebra finches
Source: PLoS One. 2026 Mar 5;21(3):e0343886. doi: 10.1371/journal.pone.0343886 (PMC12962475; doi:10.1371/journal.pone.0343886)
Supplement: S1 Table — (DOCX) [file pone.0343886.s002.docx]

**S2 Table. Shows raw time spent (sec) on each long (long path-length), short (short path-length), and home (neutral) arms during 300 sec pre-trial, trial and post-trial**. Also shows mean and standard deviation (SD) for proportion of time spent on each arm.

| **Bar # (ID Bird)** | **Pre** | | | **Trial** | | | **Post** | | |
| --- | --- | --- | --- | --- | --- | --- | --- | --- | --- |
|  | **Long** | **Short** | **Home** | **Long** | **Short** | **Home** | **Long** | **Short** | **Home** |
| Bar1 | 108.27 | 99.36 | 92.37 | 174.05 | 61.19 | 64.76 | 93.34 | 105.21 | 101.45 |
| Bar2 | 64.96 | 201.4 | 33.64 | 178.55 | 84.59 | 36.86 | 59.71 | 233.9 | 6.39 |
| Bar3 | 0 | 94.11 | 205.89 | 111.76 | 41.31 | 146.93 | 0 | 255.46 | 44.54 |
| Bar4 | 81.99 | 189.52 | 28.49 | 148.79 | 106.16 | 45.05 | 46.25 | 234.95 | 18.8 |
| Bar5 | 53.19 | 152.02 | 94.79 | 185.12 | 87.49 | 27.39 | 193.2 | 77.4 | 29.4 |
| Bar6 | 118.78 | 128.21 | 53.01 | 180.62 | 35.16 | 84.22 | 207.71 | 21.72 | 70.57 |
| Bar7 | 4.63 | 274.45 | 20.92 | 107.27 | 144.61 | 48.12 | 180.59 | 31.28 | 88.13 |
| Bar8 | 49.19 | 150.57 | 100.24 | 84.43 | 172.82 | 42.75 | 33.85 | 168.03 | 98.12 |
| Bar9 | 46.25 | 184.39 | 69.36 | 113.84 | 108.09 | 78.07 | 195.94 | 36.52 | 67.54 |
| Bar10 | 127.4 | 170.45 | 2.15 | 173.7 | 116.06 | 10.24 | 44.36 | 247.43 | 8.21 |
| Bar11 | 110.16 | 63.87 | 125.97 | 282.7 | 17.3 | 0 | 111 | 65.75 | 123.25 |
| Bar12 | 218.43 | 65.79 | 15.78 | 300 | 0 | 0 | 230.2 | 42.22 | 27.58 |
| Bar13 | 208.76 | 47.31 | 43.93 | 294.46 | 0 | 5.54 | 222.21 | 32.04 | 45.75 |
| Mean (SD) | 0.30 (0.21) | 0.46 (0.2) | 0.22 (0.19) | **0.59 (0.23)** | **0.24 (0.17)** | **0.15 (0.13)** | 0.41 (0.26) | 0.39 (0.30) | 0.18 (0.12) |
