# Supplementary material for: Re-examining hidden fitness: Female preferences for long-path songs in zebra finches
Source: PLoS One. 2026 Mar 5;21(3):e0343886. doi: 10.1371/journal.pone.0343886 (PMC12962475; doi:10.1371/journal.pone.0343886)
Supplement: S2 Table — (DOCX) [file pone.0343886.s003.docx]

**S3 Table. Effect sizes for repeated Measures ANOVA for time spent on each arm during Pre-trial, Trial, and post-trial**. See the end of the Supplementary for detailed ANOVA table.

| Comparisons | Effect size | Standard Error | Standard Error (proportion scale) |
| --- | --- | --- | --- |
| Group (Pre-Trial-Post) | η² = 0.17 | range (10.6, 26.1) | range (0.02, 0.06) |
| Long arm-Pre vs short arm-Pre | d = -0.73 | 33.39 | 0.08 |
| Long arm-Trial vs short arm-Trial | d = 1.58 | 33.62 | 0.08 |
| Long arm-Post vs short arm-Post | d = 0.07 | 48 | 0.12 |
