# Supplementary material for: Re-examining hidden fitness: Female preferences for long-path songs in zebra finches
Source: PLoS One. 2026 Mar 5;21(3):e0343886. doi: 10.1371/journal.pone.0343886 (PMC12962475; doi:10.1371/journal.pone.0343886)
Supplement: S3 Table — (DOCX) [file pone.0343886.s004.docx]

**S4 Table. Type M and Type S error and power for different hypothetical preference ratio and corresponding long-odds in logistic models.**

| Preference ratio | beta | Standard error | Power | Type S | Type M |
| --- | --- | --- | --- | --- | --- |
| 0.55 | 0.2006707 | 0.22 | 0.1493985 | 1.364637e-02 | 2.722482 |
| 0.60 | 0.4054651 | 0.22 | 0.4535250 | 1.576118e-04 | 1.466771 |
| 0.65 | 0.6190392 | 0.22 | 0.8034070 | 1.125215e-06 | 1.124541 |
| 0.70 | 0.8712224 | 0.22 | 0.9772573 | 1.646508e-09 | 1.013753 |
